# Does T1 slope minus cervical lordosis mismatch affect surgical outcomes of cervical laminoplasty in the absence of preoperative severe kyphosis?

**DOI:** 10.1186/s12891-022-05755-2

**Published:** 2022-08-25

**Authors:** Ryuji Sakamoto, Hideki Nakamoto, Yuichi Yoshida, Nozomu Ohtomo, Kosei Nagata, So Kato, Toru Doi, Yoshitaka Matsubayashi, Yuki Taniguchi, Sakae Tanaka, Yasushi Oshima

**Affiliations:** 1 Department of Orthopaedic Surgery, Faculty of Medicine, The University of Tokyo, 7-3-1, Hongo, Bunkyo-Ku, Tokyo, 113-8655 Japan; 2 Department of Orthopaedic Surgery, National Center for Global Health and Medicine, 1-21-1, Toyama, Shinjyuku-Ku, Tokyo, 162-8655 Japan

**Keywords:** Cervical myelopathy, Postoperative cervical alignment, OPLL, Kyphosis, Patient-reported outcome

## Abstract

**Background:**

The impact of the T1 slope minus cervical lordosis (T1S-CL) on surgical outcomes in patients with degenerative cervical myelopathy undergoing laminoplasty (LP) remain uncertain.

**Methods:**

One hundred patients who underwent cervical LP were retrospectively reviewed. Radiographic measurements included C2–C7 lordosis (CL), T1 slope (T1S), and C2–C7 sagittal vertical axis (SVA). Additionally, pain numeric rating scale, neck disability index (NDI), 12-Item Short-Form Health Survey, Euro QOL 5 dimensions (EQ5D), and Japanese Orthopedic Association score were investigated. According to past reports, T1S–CL > 20° was defined as mismatched. Then, based on T1S-CL mismatching, patients were divided into 2 groups.

**Results:**

This research understudied 67 males and 33 females with a mean age of 67 y. Preoperatively, only eight patients showed a T1S-CL mismatch. While the C2–7 Cobb angles did not change significantly after surgery, the T1 slope (T1S) angle increased, increasing the T1S-CL as a result. As we compared the clinical outcomes based on the preoperative T1S-CL mismatching, there were no significant differences between the two groups. On the other hand, the number of patients in the mismatched group increased to 21 patients postoperatively. As we compared clinical outcomes based on the postoperative T1S-CL mismatching, the postoperative NDI score and preop/postop EQ5D score were significantly worse in the mismatch group.

**Conclusions:**

Although cervical LP showed inferior outcomes in patients with postoperative T1S-CL mismatch even in the absence of severe preoperative kyphosis (> 10°), preoperative T1S-CL mismatch was not adversely prognostic.

## Background

Multiple surgical options for degenerative cervical myelopathy (DCM) have been observed, including posterior decompression and fusion, anterior decompression and fusion, then laminoplasty. As for posterior decompression, preoperative cervical kyphosis was related to poorer neurological improvements because of an insufficient posterior shift of the spinal cord after decompression surgery [1]. Additionally, although postoperative kyphotic changes occur after laminoplasty, the importance of preserving posterior supporting structures has been emphasized [2]. As the number of past papers on thoracolumbar deformity surgeries has increased, the influence of the T1 slope (T1S) on cervical spine surgery has also been attracting growing interest [3, 4]. Remarkably, the association between T1S and cervical lordosis (CL) has been advocated. Several past reports have also investigated the influence of T1S minus CL (T1S-CL) on the surgical outcomes of cervical spine surgery, because the ideal degree of CL is considered to be different, depending on the magnitude of T1S, including two reports on cervical laminoplasty [5, 6]. Besides, past reports included patients who experienced preoperative kyphosis of the cervical spine, which are considered to have affected the surgical results. Therefore, in this study, we solely focused on patients who lacked severe preoperative kyphosis (> 10°) and were considered indicative of laminoplasty. The purpose of this study was to investigate an influence of T1S-CL mismatch on surgical outcomes of cervical laminoplasty in the absence of preoperative kyphosis of > 10°.

## Methods

### Patient selection

The institutional review board of the authors institution approved the study protocols. We retrospectively reviewed 100 patients with degenerative cervical myelopathy who underwent double-door laminoplasty between 2004 and 2019 and replied to the questionnaires of patient-reported outcomes both pre- and postoperatively. Patients with preoperative kyphosis of > 10° or those without preoperative or postoperative radiographic examinations of the whole spine were excluded. Additionally, patients with rheumatoid arthritis, disc herniation, tumor, trauma, severe lumbar spinal canal stenosis, or previous surgery were also excluded.

### Data collection

Demographic data, including sex, age, and follow-up period, were collected from electronic medical records. Then, clinical data were evaluated using the recovery rating score of the Japanese Orthopedic Association (JOA), which was calculated using the method by Hirabayashi et al. [7]. Subsequently, neck pain was measured using the numerical rating scale (NRS). Patient-reported outcomes included the neck disability index (NDI), 12-Item Short-Form Health Survey (SF-12), and Euro QOL 5 dimensions (EQ5D). Next, pre-and postoperative standing lateral radiographs of the spine were obtained using the upper arms in a neutral hanging position. As for the radiographic parameters, the C2–C7 (CL) lateral Cobb angle, the T1 slope (T1S) angle, the C2–C7 SVA (C2-7SVA), and the T1S-CL were measured. The C2–C7 angle was measured, which was defined by the angle between the inferior end plate of C2 vertebra and the inferior endplate of C7 vertebra. T1-CL > 20° was defined as mismatching [5], and patients were divided into two based on their T1S-CL mismatching scores. Afterward, laminoplasty was conducted using an expansive double-door spinous process-splitting (Kurokawa) method [8]. The LP level depended on the degree of compression. Finally, patients were allowed to walk on the second day after surgery, isometric cervical muscle exercises allowed just after the operation, and collars were not routinely applied except when the patients complained of severe neck pain after surgery.

### Statistical analysis

Statistical analyses were conducted using JMP for Windows v.15.0 (JMP® 15 SAS Institute Inc., Cary, NC, USA). Univariate statistical analyses were performed using the Student’s paired and unpaired t-tests. The P-value < 0.05 was considered statistically significant. Finally, Pearson’s correlation coefficient was used to assess correlations between the preoperative and postoperative cervical parameters.

## Results

### Patients

One hundred (67 males; 33 females) patients were included in this study, including sixty patients with cervical spondylotic myelopathy (CSM) and 40 patients with ossification of the posterior longitudinal ligament (OPLL). The mean age at surgery was 66.9 ± 10.1 years. The mean follow-up period was 31.4 ± 20.9 months (Table 1).

**Table 1:** Demographic Data of Patients who Underwent LP for DCM

Number of cases	100
Age, y, mean ± SD	66.9 ± 10.1 ( range, 31–83)
Follow up period, mo, mean ± SD	31.4 ± 20.9 ( range, 12–99)
Sex (male: female), n	67: 33
Disease (CSM: OPLL), n	60: 40

*DCM* degenerative cervical myelopathy, *CSM* cervical spondylotic myelopathy, *OPLL* ossification of the posterior longitudinal ligament

### Radiographic and clinical outcomes

The preoperative and postoperative radiographic parameters were moderately to highly correlated, as shown in Table 2 (*r* = 0.59–0.78). While the C2–7 Cobb angle did not change significantly after surgery, the T1 slope (T1S) angle increased, increasing the T1S-CL as a result (Table 2). As for the clinical outcomes, all values improved postoperatively.

**Table 2:** Preop and Postop radiographic parameters and clinical outcomes

	Preop	Postop	*P* value	Correlation (Pearson’s *r*)
C2-C7 angle (degrees)	15.9 ± 11.6	14.8 ± 13.4	0.29	0.69
T1 slope angle (degrees)	24.8 ± 8.27	26.2 ± 9.26	0.02	0.78
T1S-C27 angle (degrees)	8.93 ± 9.45	11.4 ± 12.2	0.02	0.59
C2-C7SVA (mm)	25.0 ± 13.9	28.1 ± 17.0	0.02	0.69
NRS (Neck pain)	3.29 ± 3.08	2.69 ± 2.61	0.03	
PCS	24.9 ± 17.9	33.8 ± 17.9	< 0.01	
NDI	37.5 ± 17.7	26.6 ± 15.8	< 0.01	
EQ5D	0.57 ± 0.19	0.68 ± 0.18	< 0.01	
JOA score	10.8 ± 2.25	13.9 ± 2.21	< 0.01	

The bold value indicates considered significant (*P* < 0.05). *SVA* sagittal vertical axis, *NRS* numeric rating scale, *PCS* physical component summary, *NDI* neck disability index, *EQ5D* Euro QOL 5 dimensions, *JOA* Japanese Orthopedic Association

Next, we divided the patients into two groups based on their T1S-CL values. Preoperatively, only eight patients showed mismatches, namely, T1S-CL larger than 20°. Therefore, we compared the clinical outcomes based on the preoperative T1S-CL. Although no significant difference between the two groups was observed, postoperative NDI demonstrated worse scores in the mismatched group (Table 3). Alternatively, results showed a patient increase in the mismatched group to 21 patients postoperatively (Table 4). As we compared clinical outcomes based on the postoperative T1S-CL, the postoperative NDI and preop/postop EQ5D scores were significantly worse in the mismatched group (Table 4).

**Table 3:** Comparison of preoperative and postoperative clinical outcomes based on preoperative T1S-CL

		Mismatch group (*N* = 8)	Match group (*N* = 92)	*P* value
Age, y, mean ± SD		69.8 ± 6.65	66.7 ± 10.7	0.42
Follow up period, mo, mean ± SD		20.4 ± 7.7	32.4 ± 21.6	0.01
Sex (male/female), n		7/1	60/32	0.20
C2-C7 angle (degrees)	Preop	6.25 ± 6.32	16.7 ± 11.7	< 0.01
	Postop	2.88 ± 7.38	15.9 ± 13.4	< 0.01
T1 slope angle (degrees)	Preop	33.4 ± 8.70	24.0 ± 7.89	0.02
	Postop	31.4 ± 11.1	25.7 ± 9.07	0.19
T1S-C27 angle (degrees)	Preop	27.1 ± 5.46	7.35 ± 8.01	< 0.01
	Postop	28.5 ± 11.7	9.87 ± 11.2	< 0.01
C2-C7SVA (mm)	Preop	44.1 ± 8.66	23.4 ± 13.1	< 0.01
	Postop	47.8 ± 17.7	26.3 ± 16.0	0.01
NRS (Neck pain)	Preop	4.13 ± 2.90	3.22 ± 3.12	0.43
	Postop	3.88 ± 2.69	2.59 ± 2.60	0.19
PCS	Preop	23.1 ± 22.5	25.1 ± 17.7	0.77
	Postop	33.8 ± 10.9	33.8 ± 18.5	0.99
NDI	Preop	41.0 ± 14.7	37.2 ± 17.7	0.58
	Postop	37.3 ± 18.7	25.7 ± 15.4	0.06
EQ5D	Preop	0.51 ± 0.13	0.57 ± 0.19	0.41
	Postop	0.59 ± 0.14	0.69 ± 0.18	0.13
JOA score	Preop	10.4 ± 1.92	10.8 ± 2.29	0.62
	Postop	13.0 ± 3.06	14.0 ± 2.15	0.24
	JOARR	47.6 ± 35.2	48.9 ± 31.9	0.91

The bold value indicates considered significant (P < 0.05). *SVA* sagittal vertical axis, *NRS* numeric rating scale, *PCS* physical component summary, *NDI* neck disability index, *EQ5D* Euro QOL 5 dimensions, *JOA* Japanese Orthopedic Association, *JOARR* recovery rate of the JOA score

**Table 4:** Comparison of preoperative and postoperative clinical outcomes based on postoperative T1S-CL

		Mismatch group (*N* = 21)	Match group (*N* = 79)	*P* value
Age, y, mean ± SD		70.7 ± 7.18	65.9 ± 10.6	0.55
Follow up period, mo, mean ± SD		27.1 ± 19.9	32.5 ± 21.3	0.23
Sex ( male/female), n		16/5	51/28	0.32
C2-C7 angle (degrees)	Preop	8.52 ± 7.92	17.8 ± 11.8	< 0.01
	Postop	‐0.12 ± 11.2	18.8 ± 11.1	< 0.01
T1 slope angle (degrees)	Preop	26.1 ± 8.99	24.4 ± 8.15	0.45
	Postop	29.6 ± 10.9	25.3 ± 8.67	0.11
T1S-C27 angle (degrees)	Preop	17.6 ± 8.27	6.63 ± 8.45	< 0.01
	Postop	29.7 ± 8.21	6.49 ± 7.76	< 0.01
C2-C7SVA (mm)	Preop	32.7 ± 13.4	22.9 ± 13.5	< 0.01
	Postop	45.6 ± 17.5	23.4 ± 13.7	< 0.01
NRS (Neck pain)	Preop	3.62 ± 3.04	3.20 ± 3.12	0.59
	Postop	3.29 ± 2.83	2.53 ± 2.56	0.25
PCS	Preop	20.4 ± 18.4	26.1 ± 17.9	0.21
	Postop	34.5 ± 10.9	33.7 ± 19.5	0.86
NDI	Preop	40.4 ± 20.9	36.8 ± 16.5	0.41
	Postop	33.0 ± 15.8	24.8 ± 15.6	0.04
EQ5D	Preop	0.49 ± 0.22	0.59 ± 0.19	0.04
	Postop	0.61 ± 0.14	0.71 ± 0.19	0.02
JOA score	Preop	9.95 ± 2.89	10.9 ± 2.03	0.07
	Postop	13.2 ± 2.59	14.1 ± 2.10	0.09
	JOARR	44.9 ± 31.8	49.9 ± 32.2	0.55

The bold value indicates considered significant (*P* < 0.05). *SVA* sagittal vertical axis, *NRS* numeric rating scale, *PCS* physical component summary, *NDI* neck disability index, *EQ5D* Euro QOL 5 dimensions, *JOA* Japanese Orthopedic Association, *JOARR* recovery rate of the JOA score

## Discussion

Preoperative cervical kyphosis has been reported to result in poorer surgical outcomes after LP because of an insufficient posterior shifting of the spinal cord based. Therefore, anterior or posterior fixation surgery is considered in patients with preoperative kyphosis. However, even in patients with preoperative CL, previous studies have shown kyphotic alignment changes after LP, and poor prognostic parameters leading to postoperative kyphotic deformities after cervical LP have been reported [9, 10]. For example, Kim et al. [4] considered a high T1 slope a significant predictor of kyphotic alignment changes in patients after laminoplasty. They insisted that surgical trauma to the posterior musculature led to kyphotic deformity, because a high T1 slope represents the possibility of thoracolumbar deformity and a more prominent CL is required in such patients. The relationship between high T1 slope and increased CL is further supported by the fact that patients with a higher T1 slope demonstrated a higher incidence of postoperative interlaminar bony fusion in patients undergoing laminoplasty, possibly because such patients should have increased CL to maintain their head position [11]. Oshima et al. [12] reported that patients with larger C7-SVA showed poor patient-reported outcomes before and after surgery after adjusting for age using propensity scores. They also observed that although patients with a global imbalance also demonstrated cervical imbalance, which was considered the reason for the poor outcomes in such patients, the improvement of PROs was not different. As a result, they insisted on preserving the posterior supporting musculature particularly in patients with sagittal imbalance. Nonetheless, patients should have an adequate degree of CL dependent on the magnitude of thoracolumbar sagittal imbalance.

Additionally, it is reasonable to investigate the influence of T1S-CL on surgical outcomes after cervical surgery. According to past reports, an excellent T1S-CL value was proposed to be 16° in patients with spinal deformity [13]. Iyer et al. [14] have also shown that T1S-CL was correlated with decreasing preoperative NDI in patients undergoing cervical spine surgery. In patients undergoing LP, several past reports have investigated the influence of T1S-CL on surgical outcomes. For example, Chen et al. [6] investigated the impact of T1S-CL on postoperative neck pain (> 4 in VAS score) in 85 patients undergoing double-door laminoplasty. They reported that 20° of T1S-CL and 2.89 cm cervical SVA were considered cut-off values of postoperative neck pain. Rao et al. [5] similarly set the cut-off value of preoperative T1S-CL as 20° and investigated the influence on surgical outcomes after open-door laminoplasty. Consequently, they reported that patients with preoperative T1S-CL > 20° demonstrated a postoperative kyphotic deformity. Hence, these reports concluded that patients with preoperative T1S-CL > 20° should not undergo cervical laminoplasty to avoid postoperative cervical kyphotic changes. The current study divided the patients into two groups based on their T1S-CL mismatching (> 20°). Although patients in the postoperative T1S-CL mismatching group had lower EQ5D or NDI values, preoperative T1S-CL did not affect the surgical outcomes. One possible reason for the discrepancy between our study and past reports was that our study excluded cases of patients with severe preoperative kyphosis. Most of the patients had preoperatively cervical lordosis, and only 8% showed preoperative T1S-CL mismatch, which could have affected the statistical results. Nevertheless, a significant correlation between pre- and postoperative T1S-CL values was observed (*r* = 0.59). Interestingly, the results differed when we divided the patients based on their postoperative T1S-CL value. We observed that patients with T1S-CL mismatches demonstrated poorer surgical outcomes, which we consider reasonable because postoperative radiographic parameters more accurately represented the postoperative state of patients’ HRQOL. From the results of our study, we did not consider LP as contraindicative in patients with preoperative T1S-CL mismatch in case they did not have severe kyphosis of the cervical spine. Yet, considering that the correlation between pre-and postoperative T1S-CL values was relatively strong, patients with preoperative T1S-CL mismatch are proposed to develop postoperative T1-CL mismatch. Thus, surgeons should carefully preserve posterior supporting structures, such as semispinalis muscles attaching to C2 and nuchal ligaments connecting to C7.

This study focused on the degree of T1S-CL because we considered that the preoperative degree of cervical lordosis, not cervical sagittal balance, would influence the effectiveness of posterior decompression surgery. However, a T1S-CL mismatch > 20° was reported to be related to C2-C7 SVA > 40 mm, which is considered the threshold of cervical deformity [15, 16]. Indeed, several reports have also demonstrated the possible involvement of regional cervical balances in surgical LP outcomes. For example, cervical SVA was initially reported to be related to surgical results after a posterior fixation surgery of the cervical spine, showing that patients with postoperative cervical SVA of 40 mm or more had worse NDI scores. Similarly, Kato et al. reported that patients with preoperative cervical SVA of 35 mm or more expressed worse neck pain and SF-36 scores after cervical LP [17]. In their study, although cervical balance did not aggravate after surgery, cervical posture was considered to affect postoperative neck pain and HRQOL. Moreover, Sakai et al. [18] showed that sagittal cervical imbalance (center of gravity of the head > 42 mm) was the preoperative risk factor for kyphotic deformity after LP for CSM. These reports indicated surgical results based on postoperative neck pain or HRQOL, which can differ from the symptoms directly related to cervical myelopathy. Nevertheless, it should be noted that postoperative cervical kyphosis can occur after cervical laminoplasty in patients with a large cervical SVA or T1S-CL mismatch.

There are several limitations in this study. First, it is important to recognize that this study used a retrospective design. Second, the sample size was relatively small. Third, our sample lacked patients with severe cervical kyphosis (> 10°). Therefore, further prospective studies should provide sufficient evidence for treating patients with DCM.

## Conclusions

Although cervical LP showed inferior outcomes in patients with postoperative T1S-CL mismatch even in the absence of severe preoperative kyphosis (> 10°), preoperative T1S-CL mismatch was not adversely prognostic.

## Data Availability

The datasets generated and/or analyzed during the current study are not publicly available due to their containing information that could compromise the privacy of research participants but are available from the corresponding author on reasonable request.

## Abbreviations

CL: Cervical lordosis
CSM: Cervical spondylotic myelopathy
DCM: Degenerative cervical myelopathy
JOA: Japanese Orthopedic Association
NDI: Neck disability index
NRS: Numeric rating scale
OPLL: Ossification of the posterior longitudinal ligament
SVA: Sagittal vertical axis
